# Whole Salivary Cotinine Levels and Interleukin 1-β Levels among Young Adults Involuntarily Exposed to Vapor from Electronic Nicotine Delivery Systems

**DOI:** 10.3290/j.ohpd.b2805483

**Published:** 2022-03-14

**Authors:** Abdulrahman M. AlMubarak, Montaser N. Alqutub, Fawad Javed, Fahim Vohra, Tariq Abduljabbar

**Affiliations:** a Assistant Professor, Department of Periodontics and Community Dentistry, College of Dentistry, King Saud University, Riyadh, Saudi Arabia. Interpreted the results and wrote the methodology, drafted and revised the manuscript.; b Associate Professor, Department of Periodontics and Community Dentistry, College of Dentistry, King Saud University, Riyadh, Saudi Arabia. Interpreted the results and wrote the methodology, drafted and revised the manuscript.; c Assistant Professor, Department of Orthodontics and Dentofacial Orthopedics, Eastman Institute for Oral Health, University of Rochester, NY, USA. Wrote the discussion, drafted and revised the manuscript.; d Professor, Department of Prosthetic Dental sciences, College of Dentistry, King Saud University, Riyadh, Saudi Arabia. Performed the statistical analysis and interpreted the results, drafted and revised the manuscript.; e Professor, Department of Prosthetic Dental sciences, College of Dentistry, King Saud University; Research Chair for Biological Research in Dental Health, College of Dentistry, Riyadh, Saudi Arabia. Conceived and supervised the study, wrote the discussion, drafted and revised the manuscript.

**Keywords:** cotinine, electronic nicotine delivery systems, interleukin 1 beta, unstimulated whole saliva, vaping

## Abstract

**Purpose:**

To the assess whole salivary cotinine and interleukin 1β (IL-1β) levels among individuals involuntarily exposed to vapor from electronic nicotine delivery systems (ENDS) (test group) and unexposed individuals (control group).

**Materials and Methods:**

Demographic data and information related to ENDS vapor exposure were collected using a questionnaire. Unstimulated whole saliva samples were collected, unstimulated whole-saliva flow rate (UWSFR) was calculated, and cotinine and IL-1β levels were determined using enzyme-linked immunosorbent assay. Sample-size estimation and statistical analysis were performed. Regression analysis was performed to determine the correlation between whole salivary cotinine and IL-1β levels. Statistical significance was set at p < 0.05.

**Results:**

Forty-eight individuals (24 and 24 in test and control groups, respectively) were included. Mean ages of individuals in the test and control groups were comparable. In the test group, the mean duration for which the individuals inhaled vapor from ENDS in each session was 22.3 ± 9.5 min and they were exposed to ENDS vapor 12.2 ± 2.4 times daily. There was no difference in the UWSFR between patients in the test (0.21 ± 0.02 ml/min) and control (0.22 ± 0.04 ml/min) groups. Whole salivary cotinine (p < 0.001) and IL-1β (p < 0.001) levels were significantly higher in the test than control group

**Conclusion:**

Young adults involuntarily exposed to vapor from ENDS express elevated whole salivary cotinine and IL-1β levels. Long-term exposure to ENDS vapor may potentially predispose vulnerable populations to oral and systemic inflammatory diseases.

In the past, it was assumed that non-smokers were not subject to the deleterious health effects of smoking. Meanwhile, however, much scientific evidence has shown that involuntary tobacco smoke inhalation (ITSI) or passive smoking increases the risk of diseases such as cardiopulmonary disorders, colorectal carcinoma and respiratory diseases in vulnerable populations.^20,26,31^ With respect to oral health, ITSI disrupts the equilibrium between the host and environment,^7,28^ It is also associated with an increased expression of proinflammatory cytokines such as interleukin-1 beta (IL-1β) and IL-37 in unstimulated whole saliva (UWS),^14,19,25^ which contributes increased bone loss around teeth.^17^

Nicotine is a major addictive component in combustible and non-combustible tobacco products, such as cigarettes and snuff. Despite being aware of the negative effects of tobacco intake on overall health, it is often challenging for tobacco smokers to quit the habit, as abrupt nicotine withdrawal elicits unpleasant symptoms such as anxiety, constipation, and headache.^8,11,18^ Therefore, tobacco smokers frequently use alternatives such as electronic nicotine delivery systems (ENDS) to satisfy their bodily demand for nicotine.^27^ There is a perception that ENDS are a rather safe replacement for conventional tobacco smoking; however, studies have shown ENDS users are at a higher risk of developing oral and systemic diseases such as periodontitis and cardiovascular diseases compared with individuals not using any form of nicotinic products.^6,15,22,32^ It has been shown in vitro that inhalation of vapor produced by ENDS damages fibroblasts and results in the formation of aldehydes, reactive oxygen species and carbonyls that damage DNA and cause carbonylation of the extracellular matrix.^15^

Whole salivary cotinine levels (CL) are usually measured to verify self-reported tobacco-smoking habits. According to Mokeem et al,^24^ whole salivary CL are statistically significantly higher among ENDS users than in individuals not using tobacco in any form. However, that study reported no statistically significant difference in whole salivary IL-1β between ENDS users and control subjects.^24^ The authors suggested that this lack of statistical significance could be associated with the possibly short duration of vaping in the respective group. Moreover, involuntary exposure of ENDS vapor has been associated with an increased risk of cardiopulmonary disorders in children and adults.^29^ It is hypothesised that whole salivary cotinine and IL-1β levels are higher among individuals involuntarily exposed of ENDS vapor compared with unexposed individuals. The aim of the present study was to assess whole salivary cotinine and IL-1β levels among young adults involuntarily exposed to vapor from ENDS vs unexposed individuals.

## Materials and Methods

### Ethics Guidelines

The present study was performed following guidelines recognised by the Declaration of Helsinki as revised in 2013 for experiments involving human patients. All participants were informed that they could withdraw their participation at any stage of the investigation without consequences. The study was performed at a specialist dental center and ethical approval was acquired by the specialist research review board.

### Study Design

This is a cross-sectional observational study which assessed CL and IL-1β levels in unstimulated WS samples collected from individuals who were or were not routinely exposed to environmental vapor emitted by ENDS.

### Exclusion Criteria

Pregnant and/or lactating females as well as individuals using combustible or non-combustible tobacco products were not asked to participate. Individuals with oral diseases such as periodontitis and systemic diseases, including but not limited to self-reported prediabetes, diabetes mellitus, respiratory diseases, acquired immune deficiency syndrome, respiratory diseases, hepatic and renal disorders, and cardiovascular diseases, were excluded. Individuals currently using or those who reported to have used medications such as probiotics, steroids, non-steroidal anti-inflammatory drugs, bisphosphonates or antibiotics were also excluded.

### Study Groups

Individuals in the test group had daily exposure to vapor from ENDS for at least 5 continuous minutes for at least the past 12 months. The control group comprised individuals who reported to have never consumed any form of tobacco product and/or had exposure to ITSI and ENDS vapor.

### Questionnaire

Information about age, sex, daily frequency (number of times per day) of exposure to ENDS vapor and duration (in minutes) per exposure was collected using a questionnaire. Patients in the test group were also asked whether a relationship (such as parents, guardians, husband, wife, brother, sister, and/or boy-/girlfriend) existed between them and individuals using ENDS. The questionnaire was administered to all participants by the principal investigator (TA).

### Collection of Unstimulated WS

The unstimulated WS samples were collected using a standard technique as described in previous studies.^13,24^ All unstimulated WS samples were collected for 5 min by a calibrated investigator (MNA; Kappa score 0.88) who was blinded to the study groups. The whole-saliva flow rate (WSFR) was determined and recorded in ml/min. All samples were immediately centrifuged at 8000 rpm for 5 min in a cold room at 4°C, and the collected supernatant was frozen at -80°C. All samples were assessed for whole salivary cotinine and IL-1β levels within 48 h of collection.

### Assessment of Whole Salivary Cotinine and IL-1β Levels

Whole salivary CLs were determined using ELISA as a commercially available kit (Sigma-Aldrich Chemical; St Louis, MO, USA), according to the manufacturer’s instructions. The assays were performed at room temperature. Twenty µl of the standard and supernatants from unstimulated WS samples from the test and control groups were added in duplicates to the 96-well plate with 180 μI of cotinine-HRP conjugate solution. The plates were incubated for 60 min and washed with 0.01 M phosphate buffer (pH 7.4, with 0.05% Tween-201). The substrate, 150 of μI tetramethylbenzidine, was then added to each well and the plates were re-incubated for 1 h. The optical density (OD) of each well was read at 650 nm using a microplate reader (Microplate-reader, NB-Bio-Tek Instruments; Winooski, VT, USA). Whole salivary IL-1β levels were assessed in duplicate using a commercially-available ELISA kit (Quantikine1 High Sensitivity Kit, R&D Systems; Minneapolis, MN, USA) in accordance with the manufacturer’s guidelines. Samples were diluted to 1:100 in a calibrator-diluent provided in the kit. The samples were then incubated at room temperature for 20 h. Fifty mm of stop solution (2N H_2_SO_4_) was added to each well and OD was read at 490 nm with wavelength correction to 630 nm after placing the plate in a microplate reader (Microplate-reader, NB-Bio-Tek Instruments).

### Sample-size Estimation and Statistical Evaluation

Sample-size estimation was performed on data obtained from a pilot study using a software package (G*Power version 3.1.5., University of Kiel; Kiel, Germany).^9^ The results indicated that inclusion of at least 23 individuals each in the test and control groups was required to attain a power of 80% at a 0.40 f-type effect size with a type-1 error of 5%. The collected data were statistically analysed using a computer-based software package (SPSS 15.01; Chicago, IL, USA). Data normality was tested using the Shapiro-Wilk test. Group comparisons were done using the paired t-test. Logistic regression analysis was performed to determine whether a correlation existed between whole salivary cotinine and IL-1β levels. p-values < 0.05 indicated a statistically significant difference between the test and control groups.

## Results

### General Characteristics

A total of 48 participants were divided into two groups (n=24 each), test and control. There were 15 males and 9 females in the test group and 13 males and 11 females in the control group. The mean age in the test group was 25.2 ± 3.2 years, and in the control group 23.6 ± 1.5 years. In the test group, the mean duration for which the individuals inhaled vapor from ENDS in each session was 22.3 ± 9.5 min. These individuals were exposed to ENDS vapor 10.2 ± 2.4 times daily (Table 1).

**Table 1:** Demographic characteristics of the study groups

Parameters	Test group	Control group
Number of participants	24	24
Gender (male:female)	15:9	13:11
Age in years	25.2 ± 3.2 years	23.6 ± 1.5 years
Duration of exposure to vapor in minutes	22.3 ± 9.5 minutes	NA
Daily frequency of exposure to ENDS vapor	10.2 ± 2.4 times daily	NA

ENDS: electronic nicotine delivery systems; NA: not applicable.

### Whole-Saliva Flow Rate and Cotinine and IL-1β Levels

There was no statistically significant difference in the WSFR between patients in the test (0.21 ± 0.02 ml/min) and control (0.22 ± 0.04 ml/min) groups. Whole salivary cotinine (p < 0.001) and IL-1β (p < 0.001) levels were statistically significantly higher among patients in the test than in the control group (Table 2).

**Table 2:** Whole-saliva flow rate, cotinine and IL-1β levels

Parameters	Test group	Control group
Saliva flow rate (ml/min)	0.21 ± 0.02 ml/min	0.22 ± 0.04 ml/min
Cotinine levels (ng/ml)	17.5 ± 3.1 ng/ml*	0.17 ± 0.006 ng/ml
Interleukin-1 beta (pg/ml)	26.2 ± 6.4 pg/ml*	0.12 ± 0.005 pg/ml

*Compared with the control group (p < 0.001).

### Correlation of Whole Salivary Cotinine and IL-1β Levels with Duration of Exposure and Daily Frequency of Exposure

**Fig 1:**
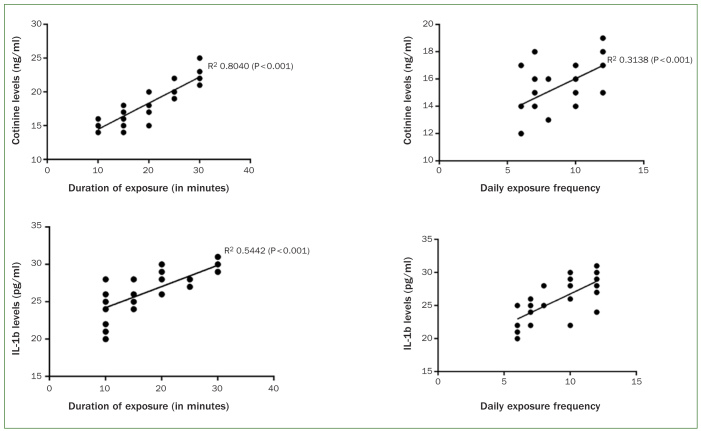
Correlation of whole salivary cotinine and IL-1β levels with the duration of exposure (in minutes) and daily frequency of exposure to emissions from electronic nicotine delivery systems.

**Fig 2:**
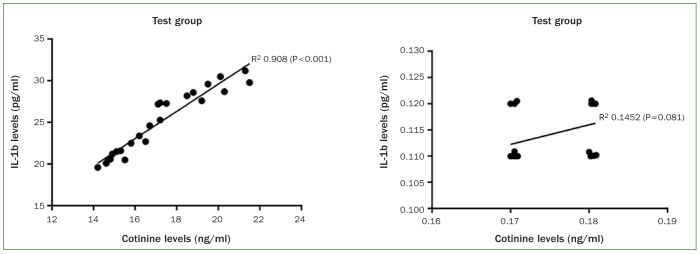
Correlation between the expression of whole salivary CL and IL-1β in the test and control groups

Whole salivary CL (p < 0.001) and IL-1β (p < 0.01) were statistically significantly correlated with duration of exposure (in minutes) and daily frequency of exposure to vapor from ENDS (Fig 1). In the test group, there was a statistically significant correlation between the expression of whole salivary CL and IL-1β (Fig 2) compared with the control group.

## Discussion

To our knowledge, this is the first study that assessed CL and whole salivary IL-1β levels among young individuals who were exposed daily to vapor emitted from ENDS. The present results support the test hypothesis, as whole salivary IL-1β and CL were statistically significantly higher in the test (exposed to vapor from ENDS) than in the control participants (never exposed to second-hand/passive smoking and/or ENDS vapor).

It has been reported that nicotine induces pathological alterations in tissues, including those of the oral cavity.^30^ According to an in vitro study,^30^ nicotine up-regulates the production of IL-1β in human periodontal ligament cells. Mahabee-Gittens et al^21^ showed that whole salivary CL >5 ng/ml are statistically significantly associated with higher whole salivary IL-1β levels. It is worth mentioning that in the present study, whole salivary CL were nearly 3x higher than those reported in the study by Mahabee-Gittens et al.^21^ Moreover, results from regression analysis showed a statistically significant association between CL and whole salivary IL-1β levels.^25^ The authors of the present study support these studies,^21,25^ as the current regression analysis confirmed a direct association between whole salivary CL and IL-1β levels, as shown in Fig 2. Moreover, our results also showed a significant association between duration of exposure to ENDS and expression of whole salivary CL and IL-1β levels (Fig 1).

Here, it is worth mentioning that all individuals using ENDS were former combustible-tobacco smokers who had quit traditional smoking and were using ENDS daily. These individuals were direct blood relatives (fathers and/or brothers) of patients in the test group. It is most probable that these individuals were using e-liquids or e-juices containing nicotine, as cotinine was identified in all unstimulated WS samples collected and assessed from patients in the test group. It is likely that ENDS users did not perceive vaping to be hazardous to their health or that of individuals around them, which allowed them to use ENDS ad libidum even in the presence of others. The results of recent studies showed that ENDS users considered vaping to be not as hazardous to health as traditional tobaccosmoking.^2,12,23^ However, abundant evidence from clinical and in vitro studies have shown that use of ENDS is not hazard-free and may have damaging effects on vital organs, including cardiovascular and respiratory systems and oral tissues.^3-5^ This suggests that there is a dire need to inform and educate the public that ENDS are by no means a safe alternate to smoking, and that environmental exposure to vapor from ENDS may induce inflammation in the long term. Community-based health awareness programs may be helpful in this regard.

There are a number of limitations inherent in the present study. Firstly, the objectives were set to determine the association between salivary IL-1β and CL in subjects involuntarily exposed to ENDS vapor. In other words, clinical oral examinations (such as periodontal status assessment) and microbiological evaluations were not performed. It has been reported that individuals exposed to ITSI present with higher probing depths and loss of clinical attachment; however, there was no statistically significant difference in clinical periodontal parameters between such individuals and non-smokers.^16^ However, ITSI has been associated with increased oral colonisation by microbes such as *Treponema denticola, Candida* and *Porphyromonas gingivalis*.^1,10^ This suggests that clinical periodontal parameters are poor and oral colonisation of periodontopathogenic microbes is increased among individuals involuntarily exposed to ENDS vapor. In this context, it cannot be maintained that exposure to ENDS vapor is safe or hazard-free. Further studies are needed in this regard. Another major obstacle in the present study was to reach consensus regarding a precise definition for patients in the test group (individuals who were exposed to and inhaled ENDS vapor on a daily basis). To our knowledge, there is no globally standardised definition for “passive vaper” or “passive smoker”. The authors suggest that further studies should be performed to reach a consensus on this.

## Conclusion

Young adults involuntarily exposed to vapor from ENDS exhibit raised whole salivary CL and IL-1β levels. Long-term exposure to ENDS vapor may potentially predispose vulnerable populations to oral and systemic diseases.
